# Plasma Soluble CD146 as a Potential Diagnostic Marker of Acute Rejection in Kidney Transplantation

**DOI:** 10.3389/fmed.2020.531999

**Published:** 2020-11-25

**Authors:** Jun Liao, Qian Fu, Wenfang Chen, Jun Li, Wenhui Zhang, Huanxi Zhang, Yifang Gao, Shicong Yang, Bowen Xu, Huiting Huang, Jiali Wang, Xirui Li, Longshan Liu, Changxi Wang

**Affiliations:** ^1^Organ Transplant Center, The First Affiliated Hospital of Sun Yat-sen University, Guangzhou, China; ^2^Department of Pathology, The First Affiliated Hospital of Sun Yat-sen University, Guangzhou, China; ^3^Guangdong Provincial Key Laboratory on Organ Donation and Transplant Immunology, Guangzhou, China; ^4^Department of Nephrology, The First Affiliated Hospital of Sun Yat-sen University, Guangzhou, China

**Keywords:** kidney transplantation, acute rejection, biomarker, melanoma cell adhesion molecule, endothelial dysfunction

## Abstract

Previous studies have implicated the role of CD146 and its soluble form (sCD146) in the pathogenesis of inflammatory diseases. However, the association between CD146 and acute rejection in kidney transplant patients remains unexplored. In this study, fifty-six patients with biopsy-proved rejection or non-rejection and 11 stable allograft function patients were retrospectively analyzed. Soluble CD146 in plasma was detected in peripheral blood by enzyme linked immunosorbent assay (ELISA), and local CD146 expression in graft biopsy was detected by immunohistochemistry. We found that plasma soluble CD146 in acute rejection recipients was significantly higher than in stable patients without rejection, and the biopsy CD146 staining in the rejection group was higher than that of the non-rejection group. Multivariate analysis demonstrated soluble CD146 as an independent risk factor of acute rejection. The area under the receiver operating characteristic curve (AUC) of sCD146 for AR diagnosis was 0.895, and the optimal cut-off value was 75.64 ng/ml, with a sensitivity of 87.8% and a specificity of 80.8%, which was better than eGFR alone (*P* = 0.02496). Immunohistochemistry showed CD146 expression in glomeruli was positively correlated with the Banff-g score, and its expression in tubules also had a positive relationship with the Banff-t score. Therefore, soluble CD146 may be a potential biomarker of acute rejection. Increased CD146 expression in the endothelial or tubular epithelial cells may imply that endothelial/epithelial dysfunction is involved in the pathogenesis of immune injury.

## Introduction

Kidney transplantation is a preferred treatment for patients with end stage renal disease (ESRD), improving quality of life and survival more so than dialysis (1). While current immunosuppressants have improved the short-term outcomes of kidney transplantation, long-term allograft loss remains a significant conundrum. Acute rejection (AR) as a result of alloimmune injury due to individual genetic differences and inadequate immunosuppression occurs in 10–20% of kidney transplant recipients and could cause allograft dysfunction. Repeated rejection episodes undoubtedly aggravate long-term renal allograft survival (2, 3). Early detection and intervention of ongoing allogeneic immune response may hinder development of rejection, alleviate immunological lesions, sustain renal function, and improve long-term graft survival. The early monitoring strategy prevents the need for later costly treatment of rejection and subsequent allograft dysfunction and provides more cost-effective benefits. In current clinical practice, acute rejection is suspected when renal function parameters such as serum creatinine and urine protein are abnormal or unstable. Renal allograft biopsy is then implemented as a gold-standard procedure to determine the diagnosis of rejection. The change of renal function parameters often lags behind the ongoing immune response and pathological lesions. Renal biopsy, as an invasive technique, may lead to surgical complications, such as bleeding, hematuria, perirenal hematoma, arteriovenous fistulas, and even graft loss (4–6), limiting its clinical usage as a consecutive monitoring tool. The needs of non-invasive or less invasive biomarkers which are necessary to discriminate rejection early from other disorders causing graft dysfunction are unmet in the field of kidney transplantation.

CD146, known as melanoma cell adhesion molecule (MCAM), is an integral membrane glycoprotein, which belongs to the immunoglobulin superfamily. Different from other widely expressed cell adhesion molecules, its expression is mainly limited to endothelial cells and pericytes (7). Besides, there are about 1–2% of lymphocytes that are detected as CD146-positive. CD146 on lymphocytes enhances production of proinflammatory cytokines and prompts inflammation (8, 9). The soluble form of CD146 (sCD146) derives from the shedding of membrane CD146 (10), and has been detected in the supernatant of endothelial cell culture medium and in the peripheral blood of patients and healthy population. Membrane CD146 and soluble CD146 can both function in cell-cell conjunction and vessel integrity, and are associated with cell signaling, migration, proliferation, differentiation, and angiogenesis (11, 12), as well as the pathogenesis of multiple illnesses including autoimmune diseases (13–15), tumors (16–19), acute heart failure (20–22), and ischemic disorders (23, 24).

A previous study found a significant increase of sCD146 in patients with chronic renal failure (CRF) compared to the heathy control, and this increase was correlated with elevated endothelial expression of CD146 in renal biopsies. It was indicated CD146 reflected the degree of endothelial dysfunction which is one of the critical changes in CRF (25). In a cohort of kidney transplantation, Malyszko and his colleagues found circulating sCD146 was higher in recipients with coronary artery disease (CAD), and this phenomenon was more apparent in patients with lower renal allograft function (26). Studies have shown the activation and injury of endothelial cells is an essential event in the initial of either T cell-mediated rejection (TCMR) or antibody-mediated rejection (ABMR). To date, clinical implication of CD146 in monitoring acute rejection after kidney transplantation has not been well-explored. We hypothesize circulating sCD146 or CD146 expression in renal allografts increases in kidney transplant patients with acute rejection. In this study, plasma sCD146 and CD146 expression in allograft biopsies of renal transplant recipients were examined to evaluate the capability of sCD146 as a less invasive biomarker of acute rejection.

## Patients and Methods

### Study Subjects

Kidney transplantation recipients who received indicated allograft biopsies from May 2016 to May 2019 in Organ Transplant Center, The First Affiliated Hospital of Sun Yat-sen University were included in this study. A total of 143 patients who underwent a renal allograft biopsy and had blood samples were included in the study. Patients with the following conditions were excluded: (1) systematic autoimmune diseases including systemic lupus erythematosus (SLE), antineutrophil cytoplasmic antibody (ANCA) glomerulonephritis, inflammatory bowel diseases (IBD), Crohn's disease and multiple sclerosis, etc; (2) diabetes mellitus; (3) active infection; (4) tumors; (5) no peripheral blood samples available before the day of biopsy. We also excluded the mixed rejection in order to more clearly figure out whether there is a difference between two types of rejection. In practice, 56 patients who met the criteria were included in the cohort analysis. Furthermore, we also enrolled 11 renal transplant patients from a surveillance program after kidney transplantation, who visited the outpatient clinic of our center at the same period, as a control group reflecting postoperative normal state. These controls had a stable allograft function, and no history of kidney diseases, diabetes, cardiovascular events, or autoimmune disorders. Hypertension, if present, was treated with a maximum of one class of antihypertensive drugs.

This study was approved by the institutional ethics committee and was conducted according to the standards of the Declaration of Helsinki. Informed consent was obtained from all subjects.

Clinical and laboratory data was collected including blood count, blood urea nitrogen, serum creatinine, serum electrolytes (potassium, sodium, and calcium), urine analysis, immunosuppression (calcineurin inhibitor trough level), and donor specific antibody (DSA). Estimated glomerular filtration rate (eGFR) was calculated according to MDRD formula (27).

### Diagnosis of Acute Rejection

All biopsies were reviewed by two independent pathologists according to the Banff classification (28, 29). Acute rejection includes antibody-mediated rejection (ABMR) and T cell-mediated rejection (TCMR). Briefly, the diagnostic criteria of acute ABMR are (1) histologic evidence of acute tissue injury, including one or more of the following: microvascular inflammation, intimal or transmural arteritis, acute thrombotic microangiopathy, and acute tubular injury; (2) evidence of antibody interaction with vascular endothelium, including at least one of the following: linear C4d staining in peritubular capillaries, at least moderate microvascular inflammation, and increased expression of gene transcriptions in the biopsy tissue indicative of endothelial injury; (3) serologic evidence of donor-specific antibodies. Acute TCMR is defined as significant interstitial inflammation and foci of moderate or severe tubulitis and/or different levels of intimal arteritis.

### Examination of Plasma sCD146 With Enzyme Linked Immunosorbent Assay (ELISA)

Peripheral blood samples were collected in plastic tubes containing ethylenediaminetetraacetic acid (EDTA) and were centrifuged at 3,000 rpm for 15 min. The supernatants were stored at −80°C until they were analyzed. Repeated thawing and freezing of the blood samples were forbidden. At the day of testing, the samples were simultaneously thawed and analyzed in triplicate. The concentration of sCD146 was determined by ELISA (RayBio® Human MCAM ELISA Kit, RayBiotech, USA) according to the manufacturer's instructions.

### Detection of CD146 Expression in Allograft Biopsies With Immunohistochemistry (IHC) Staining

CD146 immunohistochemistry staining was only performed in patients with biopsy, excluding the stable controls. Sections from formalin-fixed and paraffin-embedded tissues were dewaxed in xylene and ethanol. After antigen retrieval and endogenous peroxidase blocking, the sections were incubated with primary anti-CD146 antibody (rabbit monoclonal antibody; Abcam, Cambridge, UK), then were washed with phosphate buffered saline (PBS) before application of the anti-rabbit secondary antibody. Following 3 washes with PBS, the sections were developed with 3,3′-diaminobenzidine (DAB), and then counterstained with hematoxylin and eosin (H&E).

CD146 staining was calculated with a semi-quantitative score, as previously described (30, 31). The method, scoring the extent of CD146 staining in renal glomeruli and tubules, was as follows: Under a 200-fold microscopic field of view, 5 microscopic visions were randomly selected. In each vision, we estimated each glomerulus as follows: score 0, absence of specific staining; score 1, <25% area has specific staining for CD146; score 2, 25–50%; score 3, 50–75%; and score 4, >75%, and calculated the arithmetic average of the glomerular scores of one vision. The staining of tubular compartment was also scored on a scale of 0–4, using the same method. Then the mean value of the glomerular and tubular scores in 5 random visions were regarded as the semi-quantitative score of the glomerular and tubular CD146 staining in one patient. Representative PSAM staining of ABMR, TCMR and IF/TA are shown in Supplement Figure 1.

### Statistical Analysis

Continuous variables were described as mean and standard deviation, or median (interquartile range), as appropriate after testing for normality using the Shapiro-Wilk test. Categorical variables were described as number (percentage). Differences between the two groups were assessed with *t*-test, Wilcoxon rank sum test, or Fisher's exact test. One-way analysis of variance (ANOVA) with Tukey's multiple comparisons test or Kruskal-Wallis test with Dunn's multiple comparisons test were used when more than two groups were compared. The correlation of sCD146 and eGFR was assessed by Pearson test. Receiver operating characteristic curve (ROC) analysis of plasma sCD146 level and eGFR, and calculation of the corresponding area under the curve (AUC) was performed. The difference between two AUCs was compared using the Delong test. The optimal cut-off point was defined by the Yoden index. Logistic regression analysis was used to assess the risk factors for acute rejection. A value of *P* < 0.05 was considered to indicate statistical significance. Internal validation was performed using 2000-bootstrap resamples and the optimism-corrected AUC was also calculated. The seed for randomization was 20200101. All statistical analyses were performed using GraphPad Prism (v.7.04) and R software (v.2.10.1).

## Results

From May 2016 to May 2019, 56 patients, who met the inclusion and exclusion criteria and had matched blood samples, and 11 outpatients with stable allograft function were included in this study. The active rejection subgroup contained ACMR (*n* = 21) and TBMR (*n* = 20). The non-rejection cohort contained interstitial fibrosis and tubular atrophy (IF/TA, *n* = 7), calcineurin inhibitors (CNIs) nephrotoxicity (*n* = 4), and normal tissue (*n* = 4). The indication of biopsy included elevated serum creatinine (*n* = 37), proteinuria (*n* = 10), or both (*n* = 9). Patient characteristics are summarized in Table 1. Rejection patients had a mean age of 38.2 ± 10.7 years; 82.9% of rejection patients were male. At baseline, they were 245 [IQR, 149-1506] days after transplantation, had a mean eGFR of 43.9 ± 18.5 mL/min/1.73 m^2^, a median urinary protein excretion of 0.27 [IQR, 0.16–0.72] g/24 h. Non-rejection patients had a mean age of 32.1 ± 8.1 years; 73.3% patients were male. They were 430 [IQR, 163.5-2811] days after transplantation, had a mean eGFR of 56.6 ± 25.4 mL/min/1.73 m^2^, a median urinary protein excretion of 0.29 [IQR, 0.21–0.49] g/24 h stable controls had a mean age of 40.6 ± 10.3 years; 54.5% patients were male. They were 196 [IQR, 171–587] days after transplantation, had a mean eGFR of 74±22.6 mL/min/1.73 m^2^, a median urinary protein excretion of 0.17 [IQR, 0.1-0.21] g/24 h. All patients were primary transplant recipients, and received calcineurin inhibitors, mycophenolate, and prednisone as maintenance immunosuppressive therapy.

**Table 1:** Characteristics of kidney transplantation patients.

**Clinical Characteristics**	**Patients with biopsy**		**Stable patients**
	**Rejection**	**Non-rejection**	
Number of patients	41	15	11
Age at enrollment, y	38.2 ± 10.7	32.1 ± 8.1	40.6 ± 10.3
Men, *n* (%)	34 (82.9)	11 (73.3)	6 (54.5)
Post-transplant time d (IQR)^a^	245 (149, 1506)	430 (163.5, 2811)	196 (171, 587)
Donor type, *n* (%)			
Deceased donor	25 (61)	10 (66.7)	6 (54.5)
Living donor	16 (39)	5 (33.3)	5 (45.5)
Donor age, y	43.6 ± 15.4	42.3 ± 15.4	38.1 ± 17.7
Donor gender, Men, *n* (%)	26 (63.4)	9 (60)	5 (45.5)
Cold ischemia time, min	7.5 ± 4.2	10 ± 5.2	6.9 ± 3.5
HLA mismatch	2.2 ± 0.8	1.9 ± 0.9	1.6 ± 0.8
DSA MFI_max_ (IQR)	5635 (4618, 7590.5)^c^	–	–
eGFR^b^ [ml^*^min^−1^^*^(1.73m^2^)^−1^]	43.9 ± 18.5^*^ ^####^	56.6 ± 25.4^*^	74.0 ± 22.6
Serum creatinine μmol/L	180.5 ± 71.5^*^ ^####^	132.2 ± 46.1	87.0 ± 14.9
Proteinuria g/24 h (IQR)	0.27^*^ (0.16, 0.72)	0.29^*^ (0.21, 0.49)	0.17 (0.1, 0.21)

Compared with the group of stable patients,

* P < 0.05.

Compared with the group of non-rejection,

#### P < 0.0001.

a IQR, interquartile range.

b eGFR, estimated glomerular filtration rate.

c *the data is derived from ABMR subgroup*.

### Soluble CD146 Level Was Higher in Renal Transplantation Patients With Rejection

**Figure 1:**
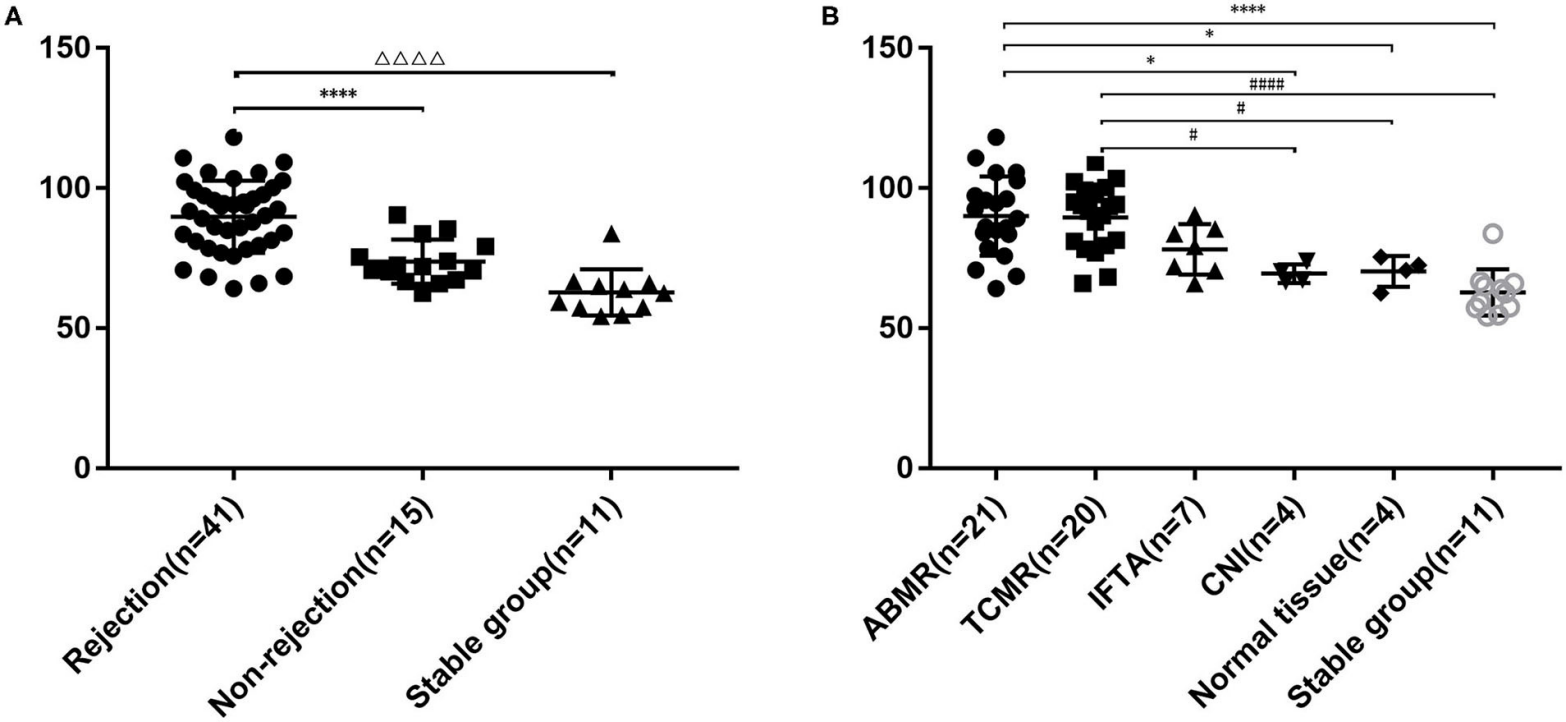
Plasma sCD146 level in kidney transplant patients with or without rejection and stable patients. **(A)** Comparison of sCD146 levels between patients with and without rejection and stable patients. The statistical differences among groups were assessed by one-way ANOVA. Statistical significance: ^ΔΔ*ΔΔ*^*P* < 0.0001, ^****^P < 0.0001. **(B)** Comparison of sCD146 levels between patients with ABMR, TCMR, IF/TA, CNI, normal allograft tissue, and stable kidney function. The statistical differences among groups were assessed by one-way ANOVA. Statistical significance: ^*^P < 0.05, ^****^P < 0.0001, ^#^*P* < 0.05, and ^####^*P* < 0.0001.

sCD146 level in the rejection group (89.8 ± 12.8 ng/ml) was significantly higher than that in the non-rejection group (73.8 ± 7.9 ng/ml, *P* < 0.0001) and control subjects (62.8 ± 8.3 ng/ml, *P* < 0.0001). The non-rejection group was slightly higher than the stable group (*P* = 0.0557) (Figure 1A). We then compared the differences of sCD146 levels between the six subgroups, i.e., TCMR, ABMR, IF/TA, CNI nephropathy, the normal tissue, and stable subjects. The sCD146 level in ABMR patients (90.0 ± 14.1 ng/ml) was higher than that in CNI nephropathy (69.5 ± 3.3 ng/ml, *P* = 0.0193), normal tissue on biopsy (70.3 ± 5.5 ng/ml, *P* = 0.0272) and stable subjects (62.8 ± 8.3 ng/ml, *P* < 0.0001), but TCMR (89.5±11.8 ng/ml, *P* > 0.9999) and IF/TA (78.2 ± 9.0 ng/ml, *P* = 0.1798). The sCD146 level of TCMR patients was higher than that of CNI (*P* = 0.0252), normal tissue (*P* = 0.0351), and control group (*P* < 0.001), but IF/TA (*P* = 0.2255) (Figure 1B).

### Plasma sCD146 Significantly Contributed to Discrimination of Acute Rejection

**Figure 2:**
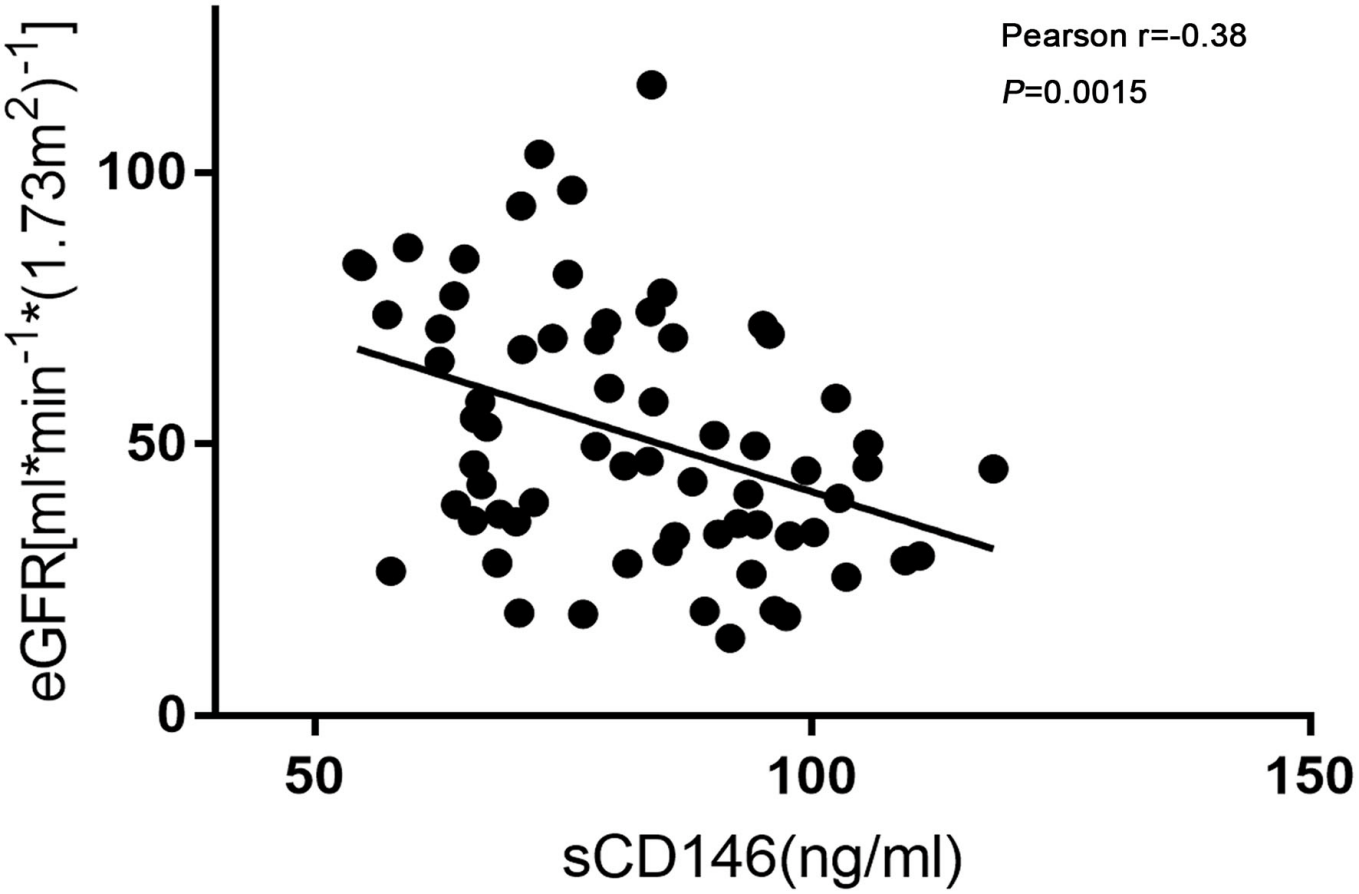
Correlation of sCD146 and eGFR. Soluble CD146 was negatively correlated with eGFR. Pearson *r* = −0.38, *P* = 0.0015.

As shown in Figure 2, sCD146 levels were negatively correlated with allograft function (Pearson *r* = −0.38, *P* = 0.0015). The diagnostic value of sCD146 for acute rejection may be affected by renal function. Thus, the logistic regression analysis was performed (Table 2). In the univariate model, sCD146 level, eGFR, and serum creatinine level were correlated with the occurrence of acute rejection. We included the parameters of which *P* < 0.10 into the multivariate logistic analysis. It was found that sCD146 level was the only independent risk factor of acute rejection.

**Table 2:** Univariate and multivariate logistic regression analysis for parameters of diagnostics of acute rejection.

	**univariate**		**multivariate**	
	**OR (95%CI)**	***P***	**OR (95%CI)**	***P***
sCD146	1.159 (1.083, 1.241)	<0.001	1.156 (1.069, 1.251)	<0.001
eGFR	0.959 (0.935, 0.984)	0.001	0.996 (0.997, 1.038)	0.853
Creatinine	1.023 (1.010, 1.036)	0.001	1.016 (0.997, 1.036)	0.108

**Figure 3:**
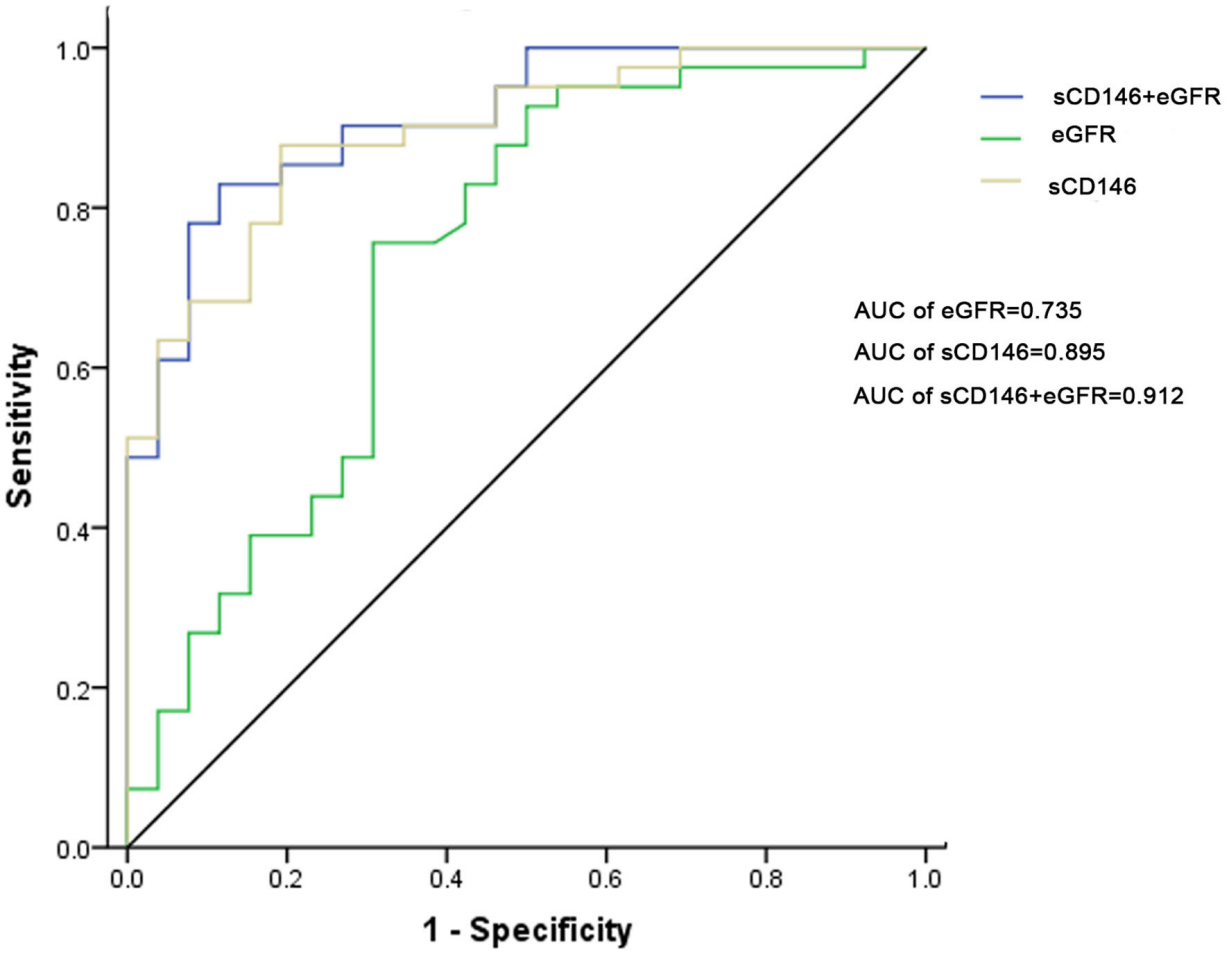
ROC curves for sCD146, eGFR, and the combination model to diagnose acute rejection. The AUC of sCD146 was higher than that of eGFR (*P* = 0.02496). The AUC of the combination model was not higher than that of sCD146 (*P* = 0.3697), but higher than that of eGFR (*P* = 0.003).

ROC curve analysis of sCD146 level and eGFR for the diagnosis of acute rejection was performed. The AUC of sCD146 was 0.895 (95% CI: 0.821–0.968; (*P* < 0.001), and the AUC of eGFR was 0.735 (95%CI: 0.605–0.865; *P* = 0.001). The optimal cut-off value of sCD146 was 75.64 ng/ml, the sensitivity was 87.8%, the specificity was 80.8%, and the corresponding positive predictive value (PPV) and negative predictive value (NPV) were 87.8 and 80.8%, respectively. Examination using the DeLong test found a significant difference between the AUC of sCD146 and the AUC of eGFR (*P* = 0.02496) (Figure 3). Furthermore, we established a combination model of sCD146 and eGFR to evaluate its diagnostic value for acute rejection, looking forward to enhancing sCD146's effectiveness. The combination model had an AUC of 0.912 (95%CI: 0.845–0.979) (*P* < 0.001), sensitivity of 82.9%, and specificity of 88.5%. The model also showed good diagnostic performance in internal validation (optimism-corrected AUC: 0.9042). The difference of the AUC between the combined model and eGFR alone (*P* = 0.003) was significant, but the combined model was not superior to sCD146 alone (*P* = 0.3697). The model also showed good diagnostic performance in internal validation (optimism-corrected AUC is 0.9042). The results suggest sCD146 significantly contributed to the diagnosis of acute rejection.

ROC curve analysis of sCD146 level and eGFR for the diagnosis of ABMR was performed, too. The AUCs of sCD146 (AUC = 0.725) and the combination model (AUC = 0.717) are better than eGFR (AUC = 0.566), respectively (*P* < 0.05). But the AUCs of sCD146 and the combination model are not significantly different (*P* > 0.05) (Supplement Figure 2).

### Local Expression of CD146 Increased in Renal Allograft Biopsies With Acute Rejection

**Figure 4:**
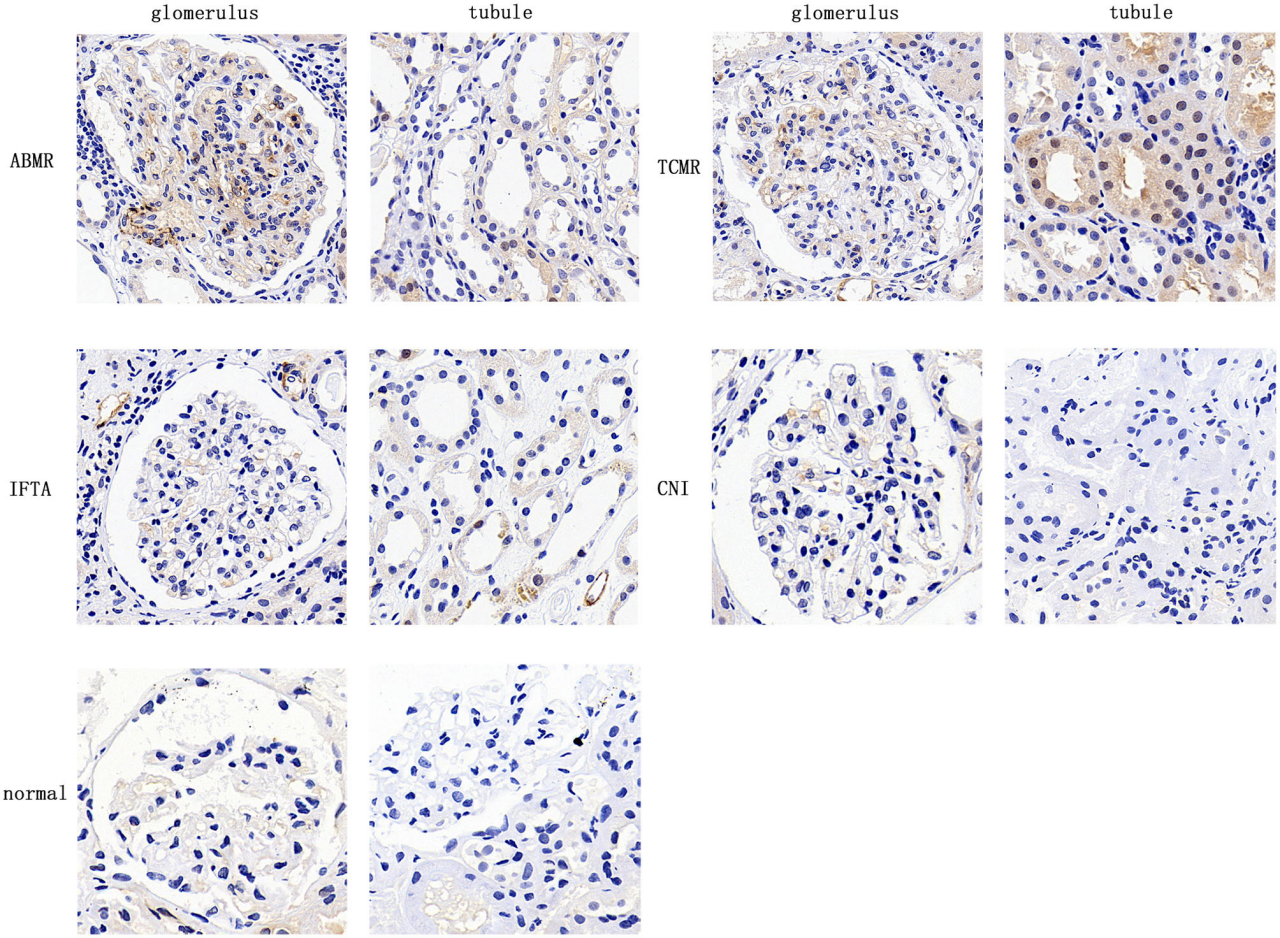
Expression of CD146 in allograft biopsy specimens of kidney transplant patients. Representative image of CD146 staining in 5 groups (×400).

**Figure 5:**
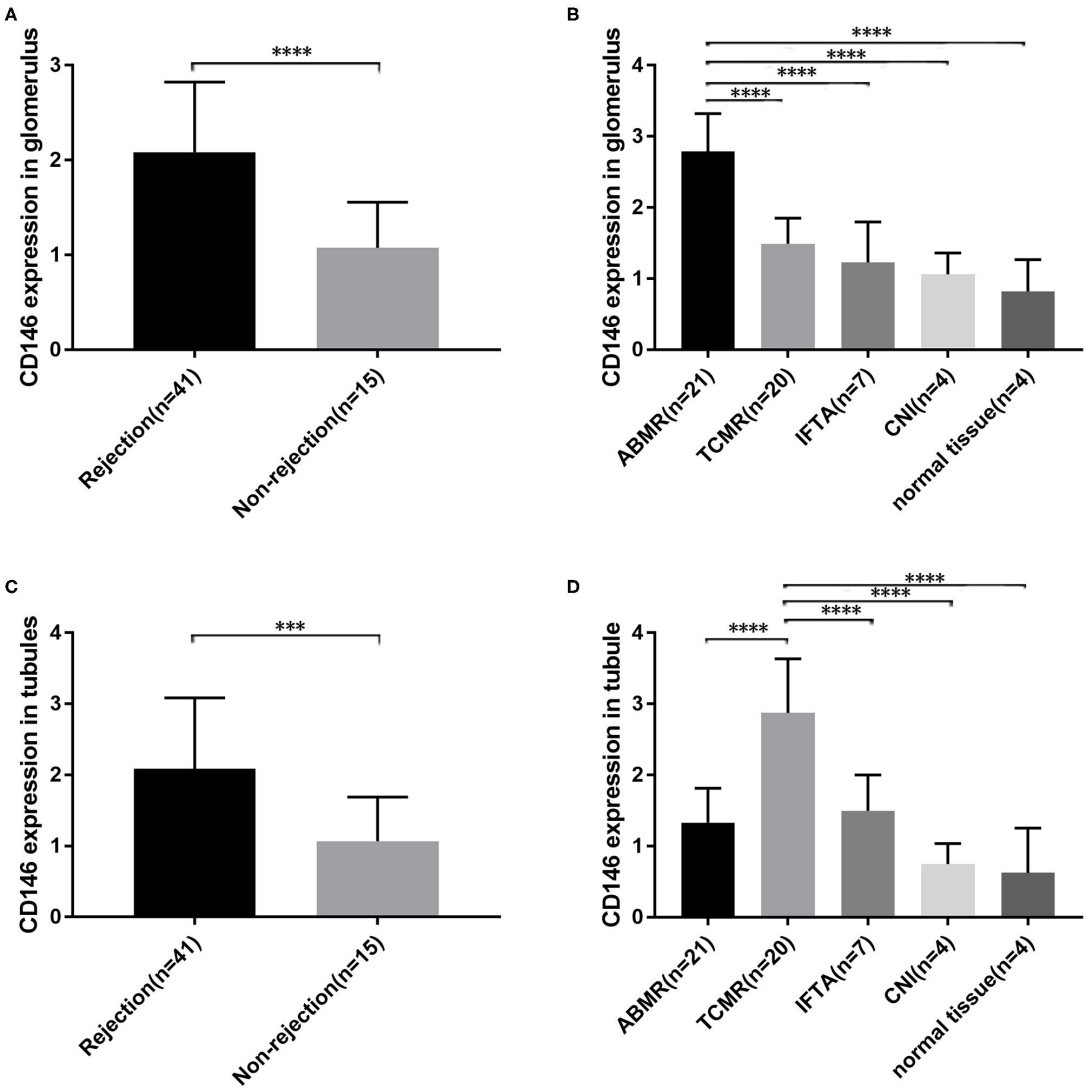
Semi-quantitative scoring of CD146 expression in different parts. **(A,C)** Semi-quantitative scoring of CD146 staining of glomerular and tubular compartments between the rejection groups and non-rejection group. **(B,D)** Semi-quantitative scoring of CD146 staining of glomerular and tubular compartments between the five subgroups. The statistical differences among groups were assessed by Student's *t*-test and one-way ANOVA. Statistical significance: ^***^P < 0.001, ^****^P < 0.0001.

CD146 expression in renal allograft biopsy sections was examined with IHC staining. Representative slide images of allograft biopsies are shown in Figure 4. The semi-quantitative scoring analysis of CD146 staining shows that the rejection group had more and stronger positive areas of CD146 expression in allograft glomeruli and tubules than the non-rejection group (Figures 5A,C). Considering the differences in the pathogenesis and pathological manifestations of ABMR and TCMR, we separated the two types of rejection and compared the expression of glomeruli and tubules, respectively (Figures 5B,D). The allograft biopsied from ABMR biopsies had more positive areas of CD146 expression in the glomerular compartment, while the range of tubular CD146 expression was relatively modest. We did not observe much positive expression in peritubular capillaries, so it was difficult to judge the condition of peritubular capillaries CD146 expression. In contrast, in patients with TCMR the staining expression of the tubular compartment was higher than that of the glomerular compartment. These data suggest that an increased, distribution-different expression of CD146 in glomeruli and tubules may be associated with these two kinds of rejections that were, respectively, characterized by glomerulitis and tubulitis.

### Allograft Expression of CD146 Was Associated With Banff Score

**Figure 6:**
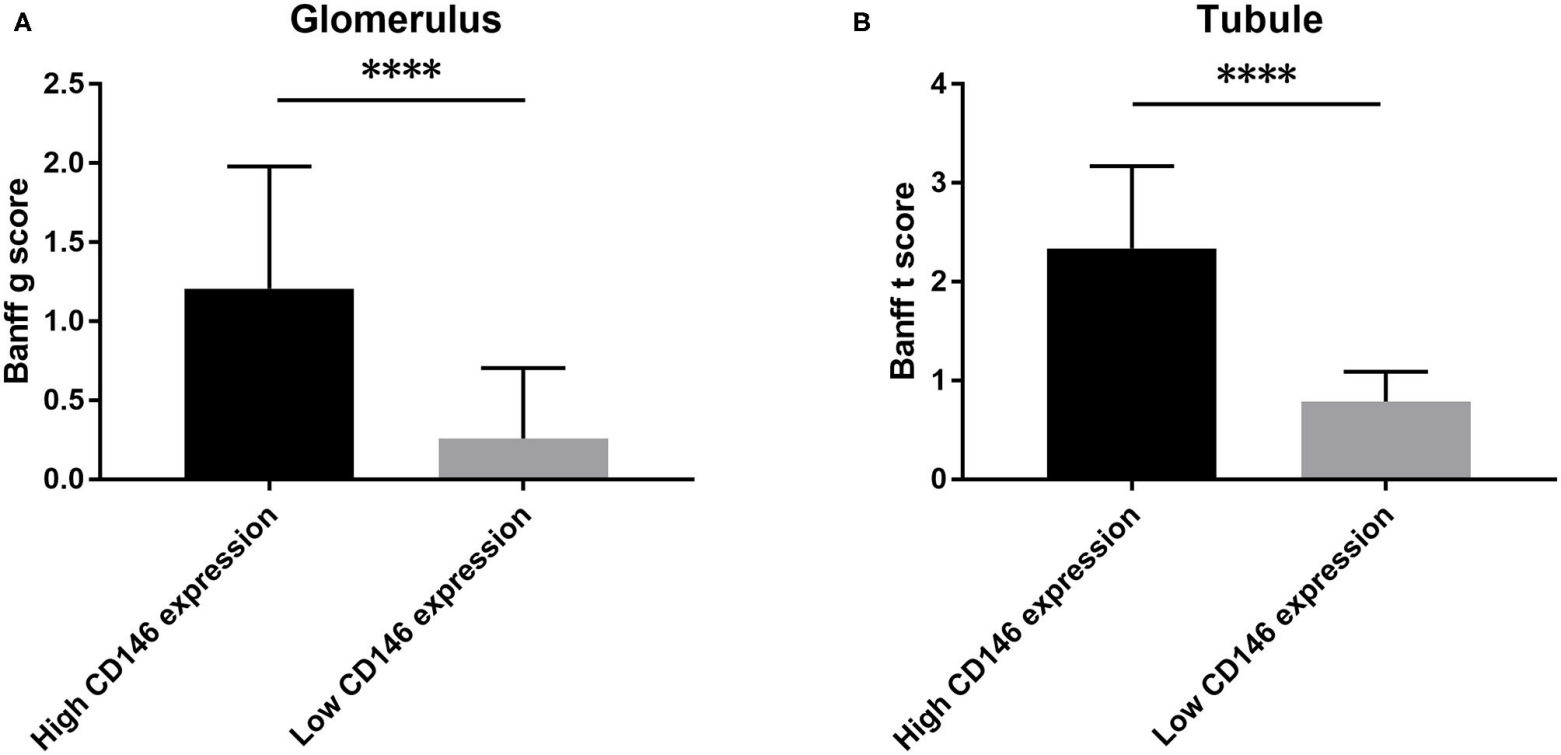
Association of CD146 expression and Banff score. **(A)** The correlation of CD146 expression in glomerulus and Banff g score. **(B)** The correlation of CD146 expression in tubule and Banff t score. Statistical significance: ^****^P < 0.0001.

The correlation of CD146 expression in renal biopsy specimens with Banff score was further analyzed. The median values of glomerular and tubular CD146 staining scores were defined as the corresponding cut-offs. The high glomerular CD146 expression was defined as glomerular semi-quantitative score ≥1.7, and the high tubular CD146 expression was defined as tubular semi-quantitative score ≥1.5. Our results showed that high glomerular CD146 expression was positively correlated with increased Banff g score (*P* < 0.0001), and high tubular CD146 expression was positively correlated with increased Banff t score (*P* < 0.0001) (Figure 6). These results suggest that CD146 expression in renal biopsy specimens may be useful for evaluating the severity of inflammatory infiltration in acute rejection.

## Discussion

The results of this study suggest that plasma sCD146 level may be useful for monitoring acute rejection in renal transplant recipients, and sCD146 might function as a pro-inflammatory marker which facilitates the development of rejection.

Prior studies have shown that CD146 and its soluble form are associated with endothelial dysfunction or injury and play a crucial role in inflammatory diseases. Bardin et al. reported that CD146 expression was increased in endothelial cells from intestinal biopsy specimens from patients with active IBD, Crohn's disease or ulcerative colitis, especially in actively inflamed areas. The results reflected the important role of CD146 in endothelial dysfunction, vascular permeability, and vessel proliferation (14). Studies of multiple sclerosis, which is an inflammatory disease of the central nervous system, showed the expression of CD146 in the blood-brain-barrier promoted the transmigration of leukocytes, effectively triggering focal inflammation (13, 15, 32). Higher sCD146 levels in the cerebrospinal fluid of patients with multiple sclerosis are also correlated with the disease severity. In the current study, we found that the level of sCD146 was higher in recipients with acute rejection, implying the allografts were in an active inflammatory phase, in accordance with the previous research results about inflammation related diseases. Endothelial dysfunction has a significant impact on the pathogenesis of acute rejection (33), so we claimed that sCD146, as a marker of endothelial dysfunction, may be useful for estimating the severity of inflammation and the seriousness of acute rejection.

When designing the study, we excluded the mixed rejection patients because of the consideration of the different mechanisms of ABMR and TCMR, which might differ the concentrations of sCD146. The main feature of ABMR is that the antigens of vascular endothelial cells are recognized by various antibodies, causing a series of subsequent rejection effects, however, TCMR involves the infiltration of mononuclear lymphocytes into renal tubules and interstitial. Unexpectedly, the plasma levels in both rejection subgroups were elevated. Three possibilities may explain the phenomenon: (1) TCMR is also involved in intimal arteritis, which can cause an increase in soluble CD146; (2) renal tubular epithelial cells are induced to express CD146 (30), which resulted in increased plasma concentration after shedding; (3) the experimental or statistical error caused by the too-small sample size.

We found that a sCD146 plasma level of 75.64 ng/ml had a specificity of 87.8% and a sensitivity of 80.8% for predicting acute rejection, with an AUC of 0.895. We further established a combination model suing sCD146 and eGFR, which had an AUC of 0.912, a sensitivity of 82.5%, and a specificity of 88.9% for discriminating acute rejection. According to our model, the detection of sCD146 alone had a high sensitivity and specificity, which meant that patients who used sCD146 level to detect whether acute rejection occurs might have relatively satisfactory diagnostic accuracy. When combined with eGFR to form a joint model, the AUC of the novel model seems to improve up to 0.912, but statistical analysis does not support this modest improvement. In patients with abnormal or unstable renal function after transplantation, sCD146 may have the potential to be a sensitive indicator for the occurrence of allograft rejection. Although allograft biopsy is still the gold standard, the detection of sCD146 level could complement the diagnostic efficiency of common clinical indicators and increase the accuracy of diagnosis of acute rejection.

We also found the staining degree of CD146 was greater in biopsy specimens of acute rejection. It is reasonable to deduce that the increased plasma sCD146 level might come from the allografts undergoing rejection by Logistic regression analysis. CD146 could make endothelial cells to remodel their cytoskeleton, facilitating certain kinds of leukocytes and small activated molecules to pass through the barriers and then infiltrate local inflammatory tissues (34). Interestingly, the distribution pattern of CD146 staining was not the same in ABMR and TCMR. In ABMR, CD146 staining was greater in the glomerular compartment, while in TCMR staining was concentrated in the tubules. In addition, glomerular CD146 staining was positively correlated with biopsy Banff g score, and tubular staining was correlated with Banff t score. These data may support the hypothesis that high expression of CD146 is associated with the severity of local inflammation.

As previously described, CD146 expression is limited to certain cells and tissues. Moreover, the normal distribution of CD146 expression will change under certain condition of diseases. Some cell types that do not initially express CD146, such as renal tubular epithelial cells, will highly express it (25). Changes of the expression pattern of CD146 in cells may imply transformation of the function and structure of these cells. In this study, the expression of CD146 was different between the TCMR and ABMR groups. There was up-regulation of CD146 in the tubular region of patients with TCMR and the glomerular region of patients with ABMR, which is consistent with the respective pathological changes—tubulitis and glomerulitis.

To our best knowledge, this is the first study to explore the relation of sCD146/CD146 and acute renal rejection. Besides the new findings of this study, there are some limitations. Firstly, this was a single center retrospective study with a relatively small sample size. Secondly, the value of sCD146 for diagnosis of acute rejection needs further external evaluation. Thirdly, the potential mechanism explaining how CD146 contributes to the acute rejection requires further investigation.

In summary, our findings suggest that sCD146 may be useful for diagnosing acute rejection episodes in renal transplant recipients. The increased expression of CD146 may reflect endothelial or tubular epithelial dysfunction in the pathogenesis of immune injury.

## Data Availability Statement

All datasets generated for this study are included in the article/Supplementary-Material.

## Ethics Statement

The studies involving human participants were reviewed and approved by the ethics committee of the first affiliated hospital of Sun Yat-sen University. The patients/participants provided their written informed consent to participate in this study.

## Author Contributions

JLia and QF conception and design, collection and assembly of data, data analysis and interpretation, manuscript writing, final approval of manuscript. WC, WZ, and SY pathological examination and analysis, final approval of manuscript. JLi, BX, and HH collection and assembly of data, data analysis and interpretation, final approval of manuscript. YG, JW, and XL laboratory experiments. HZ statistical analysis. LL and CW conception and design, financial support, data interpretation, manuscript writing, final approval of manuscript.

## Conflict of Interest

The authors declare that the research was conducted in the absence of any commercial or financial relationships that could be construed as a potential conflict of interest.

## Abbreviations

ABMR: antibody-mediated rejection
ANOVA: analysis of variance
AR: acute rejection
AUC: area under the curve
BBB: blood brain barrier
CAD: coronary artery disease
CAN: chronic allograft nephropathy
CNINT: calcineurin inhibitors nephrotoxin
CNS: central nervous system
CRF: chronic renal failure
EDTA: ethylenediaminetetra-acetic acid
ELISA: enzyme linked immunosorbent assay
ESRD: end stage renal disease
eGFR: estimated glomerular filtration rate
IBD: inflammatory bowel disease
IF/TA: interstitial fibrosis and tubular atrophy
IQR: interquartile range
MCAM: melanoma cell adhesion molecule
NPV: negative predictive value
PBS: phosphate buffered saline
PPV: positive predictive value
ROC: receiver operating characteristic curve
TCMR: T cell mediated rejection.
