# Supplementary material for: Plasma Soluble CD146 as a Potential Diagnostic Marker of Acute Rejection in Kidney Transplantation
Source: Front Med (Lausanne). 2020 Nov 25;7:531999. doi: 10.3389/fmed.2020.531999 (PMC7729194; doi:10.3389/fmed.2020.531999)
Supplement: Supplementary file 1 [file Table_1.DOCX]

**Supplement Figure 1. Representative PSAM staining of ABMR, TCMR, and IF/TA. (A)** Representative PSAM staining of ABMR (×400). **(B)** Representative PSAM staining of TCMR (×400). **(C)** Representative PSAM staining of IF/TA (×400).

**Supplement Figure 2. ROC curves for sCD146, eGFR, and the combination model to diagnose ABMR.** The AUCs of sCD146 (AUC=0.725) and combination model (AUC=0.717) are better than eGFR (AUC=0.566), respectively(*P*＜0.05). But the AUCs of sCD146 and combination model are not significantly different(*P*＞0.05).
