# Supplementary figures and images for: Plasma Soluble CD146 as a Potential Diagnostic Marker of Acute Rejection in Kidney Transplantation
Source: Front Med (Lausanne). 2020 Nov 25;7:531999. doi: 10.3389/fmed.2020.531999 (PMC7729194; doi:10.3389/fmed.2020.531999)

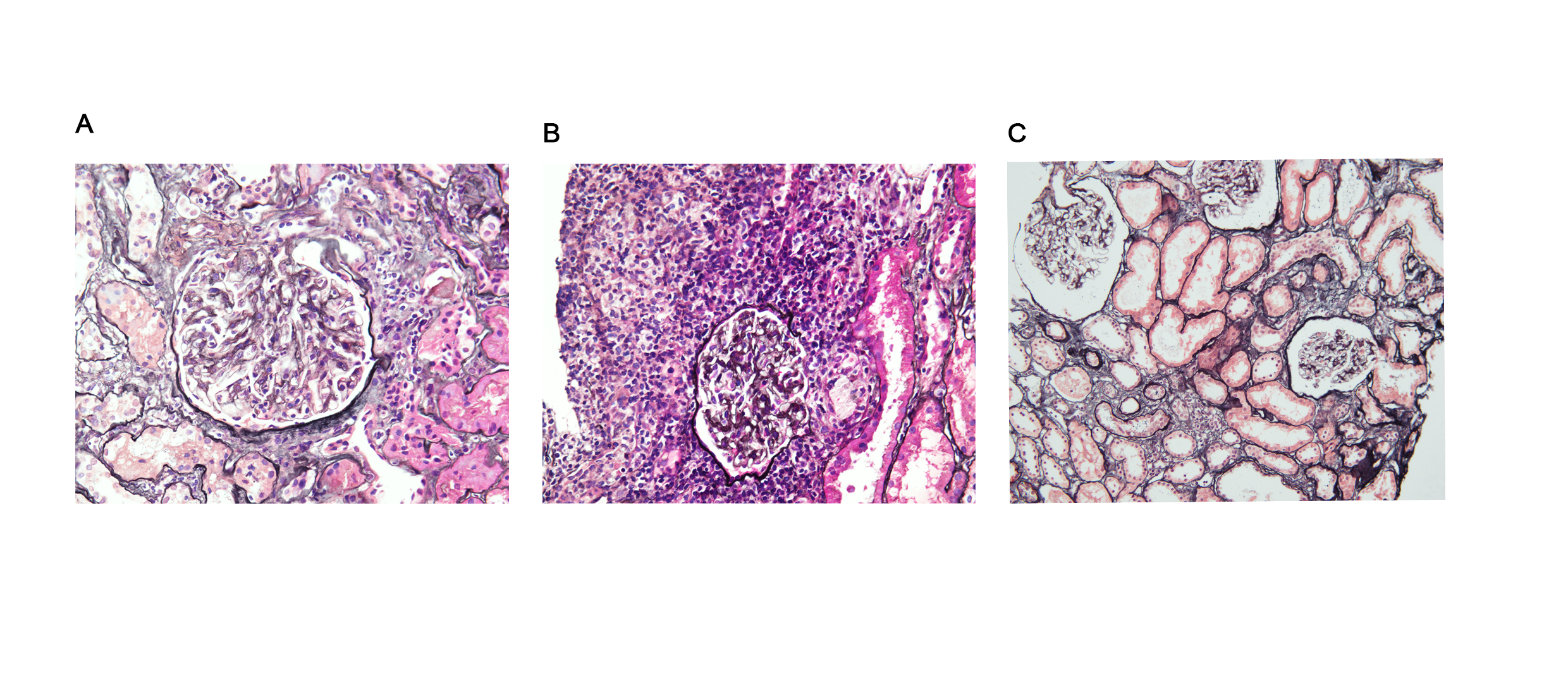

Supplement: Supplementary file 2 [file Image_1.TIF]

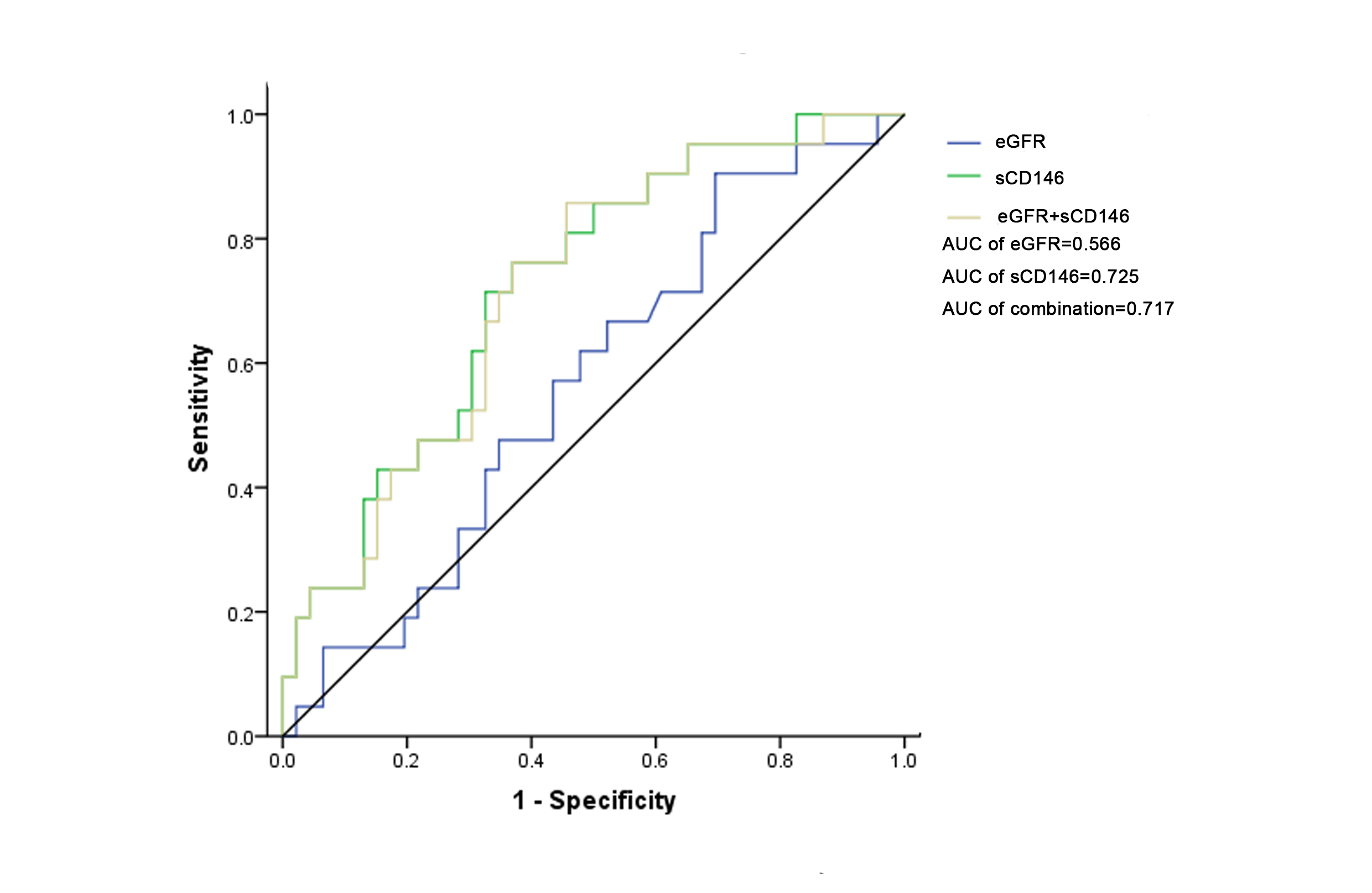

Supplement: Supplementary file 3 [file Image_2.TIF]
